# Systemic and Ocular Long Pentraxin 3 in Patients with Age-Related Macular Degeneration

**DOI:** 10.1371/journal.pone.0132800

**Published:** 2015-07-15

**Authors:** Helene Bæk Juel, Carsten Faber, Lea Munthe-Fog, Simone Bastrup-Birk, Alexander Lynge Reese-Petersen, Mads Krüger Falk, Amardeep Singh, Torben Lykke Sørensen, Peter Garred, Mogens Holst Nissen

**Affiliations:** 1 Eye Research Unit, Department of International Health, Immunology and Microbiology, University of Copenhagen, Copenhagen, Denmark; 2 Department of Ophthalmology, Glostrup Hospital, Glostrup, Denmark; 3 Laboratory of Molecular Medicine, Department of Clinical Immunology, Rigshospitalet, Copenhagen University Hospital, Copenhagen, Denmark; 4 Department of Ophthalmology, Copenhagen University Hospital, Roskilde, Denmark; 5 Faculty of Health and Medical Sciences, University of Copenhagen, Copenhagen, Denmark; Centre for Eye Research Australia, AUSTRALIA

## Abstract

Age-related macular degeneration (AMD) has been associated with both systemic and ocular alterations of the immune system. In particular dysfunction of complement factor H (CFH), a soluble regulator of the alternative pathway of the complement system, has been implicated in AMD pathogenesis. One of the ligands for CFH is long pentraxin 3 (PTX3), which is produced locally in the retinal pigment epithelium (RPE). To test the hypothesis that PTX3 is relevant to retinal immunohomeostasis and may be associated with AMD pathogenesis, we measured plasma PTX3 protein concentration and analyzed the RPE/choroid *PTX3* gene expression in patients with AMD. To measure the ability of RPE cells to secrete PTX3 in vitro, polarized ARPE-19 cells were treated with activated T cells or cytokines (interferon (IFN)-gamma and/or tumor necrosis factor (TNF)-alpha) from the basolateral side; then PTX3 protein concentration in supernatants and *PTX3* gene expression in tissue lysates were quantified. Plasma levels of PTX3 were generally low and did not significantly differ between patients and controls (P=0.307). No statistically significant difference was observed between dry and exudative AMD nor was there any correlation with hsCRP or CFH genotype. The gene expression of *PTX3* increased in RPE/choroid with age (P=0.0098 macular; P=0.003 extramacular), but did not differ between aged controls and AMD patients. In vitro, ARPE-19 cells increased expression of the *PTX3* gene as well PTX3 apical secretions after stimulation with TNF-alpha or activated T cells (P<0.01). These findings indicate that PTX3 expressed in the eye cannot be detected systemically and systemic PTX3 may have little or no impact on disease progression, but our findings do not exclude that locally produced PTX3 produced in the posterior segment of the eye may be part of the AMD immunopathogenesis.

## Introduction

Age-related macular degeneration (AMD) is the most common cause of vision loss for persons above 50 years of age in Western countries [1,2]. Though the pathogenesis remains elusive, it involves progressive atrophy of the macular retinal pigment epithelium (RPE) and subsequently photoreceptors. The prevalence of late-stage AMD increases with age from about 1% in <70 year-olds to 15% in >80 year-olds [3]. Because the elderly population is rising in industrialized countries, it is expected that the number of AMD patients will increase significantly in coming years.

The earliest hallmark of AMD is the emergence of drusen, sub-RPE deposits consisting of protein and lipids. Protein analyses of drusen from postmortem eyes have revealed a plethora of inflammatory proteins including complement factors [4–10], cytokines [11], and C-reactive protein (CRP) [8,12]. It is hypothesized that the accumulating drusen can trigger local production of inflammatory mediators and attraction of leukocytes, which would lead to an increase in inflammation and retinal stress [6]. Recent experimental studies point towards immunological and oxidative processes leading to RPE cell death and AMD [13].

The complement system is the first line of defense against microbial intruders, and important for endogenous tissue homeostasis through the opsonization of apoptotic cells and debris. It is under tight regulation by soluble and membrane-bound inhibitors, and many complement factors are expressed in the normal human retina. Interestingly, there is constitutive complement activation at the RPE/choroidal interface, and increasingly so with aging [14,15].

The pentraxin superfamily includes the classical short pentraxins CRP and serum amyloid P component (SAP), and the long pentraxins, including long pentraxin 3 (PTX3) [16]. A PTX3 plasma concentration around 2.0 ng/ml has been demonstrated in healthy individuals [17,18]. While CRP and SAP are primarily produced in the liver and released to the blood, PTX3 is produced by local tissues at sites of inflammation. This includes the RPE, where PTX3 expression is induced by inflammatory stimuli [19]. To the best of our knowledge, ocular or systemic levels of PTX3 in patients with AMD have not been reported before. However, PTX3 is reportedly increased in plasma from individuals with other inflammatory diseases, including Parkinson’s, multiple sclerosis, systemic lupus erythematosus, and sepsis [17,18,20,21].

Complement factor H (CFH) is a soluble regulator of the alternative pathway of complement and dysfunction of CFH due to several single nucleotide polymorphisms in the *CFH* gene has been implicated in the pathogenesis of AMD [22,23]. PTX3 activates the classical and the lectin complement pathways through interaction with C1q or the ficolins on cell surfaces, but it also mediates down regulation of alternative complement pathway amplification and activation by attracting CFH [24].

Based on the retinal expression of PTX3, the known interaction between PTX3 and CFH and the strong association between CFH polymorphisms and AMD, we hypothesized that retinal PTX3 production could be important for the local immunohomeostasis and that aberrant expression of PTX3 could be associated with the pathophysiology of AMD.

## Materials and Methods

### Ethics Statement

Before enrollment, informed written consent was obtained from all participants. The protocol adhered to the tenets of the Declaration of Helsinki, and approval was obtained from The Regional Ethics Committee of Zealand.

### Cases and Controls

Participants were recruited from the Department of Ophthalmology, Copenhagen University Hospital, Roskilde, Denmark. Participants were assessed for medical conditions, current medication, body mass index (BMI), and smoking habits (current [current smokers and persons reporting quitting smoking within the past year]; former smokers [persons reporting to have smoked > 100 cigarettes during their life, but none within the last year]; or never smokers). Persons with a history of malignancy, autoimmune or hematologic disease were excluded. None had received ranibizumab within the past 30 days, and all were naïve to bevacizumab and aflibercept. Cases and controls were included in parallel.

The diagnosis of AMD was based on an ophthalmoscopic fundus examination, digital fundus photography (Carl Zeiss, Jena, Germany), autofluorescence imaging, and spectral-domain optical coherence tomography (Spectralis HRA+OCT, Heidelberg Engineering, Heidelberg, Germany). After blood sampling, we performed fluorescein angiography and indocyanine green imaging (Spectralis HRA-OCT) in patients with suspected AMD. Participants were graded according to the Clinical Age-Related Maculopathy Staging System (CARMS) [25]. Participants without AMD were divided into groups of young (<60 years) or aged controls (>/ = 60 years). Part of the study population has been described previously [26,27].

### Plasma Measurements

Peripheral venous blood were drawn in heparinized tubes between 8:00 and 10:00 AM, stored at room temperature, and processed within 6 hours. For isolation of plasma, tubes were centrifuged at 1000xg for 15 minutes; plasma was isolated and stored at -80°C before analysis.

Genotyping for the CFH^Y402H^ single nucleotide polymorphism was carried out with the CFH H402 and Y402 variant detection ELISA kit (Hycult Biotech, Uden, Holland).

For the PTX3 quantification, microtiter plates (Maxisorp Nunc Immuno plates, Nalge Nunc International, Rochester, NY) were coated with 3 μg/ml anti-PTX3 antibody (moabPTX3-66) [28] in PBS overnight at 4°C. Plasma samples were diluted 1:20 in sample buffer containing 0.1% murine and bovine serum, 1% Cross-Down buffer (AppliChem, Darmstedt, Germany) and 5 mM EDTA in PBS-T to avoid heterophilic interference. Recombinant PTX3 was used as calibrator. Following incubation for 3 hours at 37°C, 2 μg/ml biotinylated detection antibody (moabPTX3-20) was added to the wells and the plates were incubated overnight at 4°C. Next day, streptavidine-horseradish peroxidase conjugate (GE Healthcare, Freiburg, Germany) diluted 1:2000 in PBS-T was added and incubated for 2 hours at 37°C. The plates were developed with TMB-sens (Kem-En-Tech Diagnostics, Taastrup, Denmark), stopped with 1 M sulphuric acid and measured at 450 nm. Between each step, wells were washed four times with PBS-T. The antibodies applied have previously been described [28]. The detection limit of PTX3 in plasma was 1.5 ng/ml and the assay has previously been shown to correlate highly with a commercially available PTX3 ELISA (26 paired samples, rho 0.8, p<0.001) [28].

High-sensitivity (hs)CRP was measured with a latex immunoassay using Architect CI8200 (Abbott Laboratories, Abbott Park, IL, USA) with a detection limit of 0.2 μg/ml.

### In silico *PTX3* gene expression analysis of human RPE/choroid tissue

A publicly available gene expression dataset was downloaded from the NCBI GEO database [29] (GSE29801). This dataset included gene expression data from RPE/choroid (extramacular RPE/choroid, n = 83; macular RPE/choroid, n = 90) and neuroretinas (extramacular neuroretinas, n = 58; macular neuroretinas, n = 60) from two cohorts of young and aged controls and patients with various stages of AMD [30]. The individual data files were merged, and expression data for beta actin (*ACTB*) and *PTX3* were extracted for analysis.

### Cell culture

The adult human RPE cell line ARPE-19 (American Type Culture Collection) was cultured on either 0.2 μm membrane inserts (discontinued; Anopore, Nalge Nunc International, Rochester, NY, for gene expression studies) or 0.4 μm membrane inserts (Falcon, BD Biosciences, Rockville, MD, for PTX3 secretion quantification) >6 weeks, until pigmented monolayers containing approximately 1.6 million cells had formed. Cells were initially cultured in Dulbecco’s modified Eagle’s medium with 10% fetal calf serum (BioWhittaker, Lonza Group Ltd, Basel, Switzerland), 300 μg/ml L-glutamine, 50 μg/ml gentamicin, and 2.5 μg/ml amphotericin B (all from Life Technologies, Carlsbad, CA). Medium was gradually changed to X-vivo 15 serum-free medium (BioWhittaker) containing 300 μg/ml L-glutamine, 100 μg/ml streptomycin, and 2.5 μg/ml amphotericin B, 2–4 weeks before experiments.

### T cell purification and RPE:T cell co-culture

T cells were purified from fresh whole blood from healthy, young volunteers by isopycnic centrifugation (Lymphoprep, Axis-Shield) and T cell negative selection (Dynal T cell negative selection kit, Invitrogen), as previously described [31]. Verbal consent to blood sampling was considered adequate by the local Ethics Committee, and was obtained. Biological specimens and all data obtained from their use for research were anonymized. Recruitment, verbal consent, and storage/use of blood specimens were carried out in accordance with the Declaration of Helsinki. T cells were added in the ratio 2.5 T cells: 1 RPE cell to the bottom or apical compartment as previously described with Dynabeads CD3/CD28 T Cell Expander (Invitrogen) [31]. This ratio was chosen to achieve cytokine concentrations in the supernatant comparable to concentrations in vivo in areas with high local T cell density. At the end of co-culture, media was gathered, and RPE cells were removed using a cell scraper. RNA was prepared as previously described [31]. A different donor was used for each independent replicate. Cell culture supernatants were diluted 1:5 or 1:10 in PBS-T with 5 mM EDTA for PTX3 protein quantification.

### Recombinant cytokines and neutralizing antibodies

Cytokines were added to the basolateral compartment in the concentration 200 ng/ml IFNγ and/or 40 ng/ml TNFα (both from R&D Systems). These concentrations were found by ELISA to yield approximately the same concentrations after 48h, as found in 48h co-culture media from RPE cells with activated T cells (data not shown). Media was collected and RNA was purified as above.

### Microarrays

The genome-wide microarrays Human Genome U133 Plus 2.0 and Human Gene 1.0 ST (both from Affymetrix) were labeled and analyzed as previously described [32]. These datasets have previously been deposited at the NCBI GEO database (GSE17938 and GSE36331).

### Statistics

Categorical demographic and clinical data were compared using the Pearson chi-square test. Plasma PTX3 concentration in patients and controls, and correlation between PTX3 and CFH^Y402H^ genotype was compared with the 1-way ANOVA. Correlation between plasma levels of PTX3 and hsCRP was analyzed with linear regression analysis. The *PTX3* gene expression in the human RPE/choroid tissue samples was analyzed with unpaired t test with Welch’s correction for unequal variances. RPE cell gene expression data were analyzed with 1-way ANOVA with Dunnett’s multiple comparison. Apical and basolateral secretion of PTX3 was analyzed separately with repeated measures 1-way ANOVA with Dunnett’s multiple comparison. All data were analyzed with SPSS version 20 for Windows (SPSS Inc., Chicago, IL) or GraphPad Prism 4 for Windows (GraphPad Software Inc., San Diego, CA). Scatter plots were prepared in GraphPad Prism. Two-sided P values < 0.05 were considered statistically significant.

## Results

We included 124 persons with AMD and 118 controls (Table 1). Of the controls, 61 were aged 60 years or older. The AMD group included 26 persons with early AMD (CARMS grade 2 or 3), 17 persons with geographic atrophy (CARMS grade 4), and 81 persons with exudative AMD (CARMS grade 5). Compared with the aged controls, the persons with AMD were older and had an increased frequency of the CFH^402H^ risk allele (Table 1).

**Table 1:** Demographics and clinical characteristics.

	Controls <60y (n = 57)	Controls = />60y (n = 61)	Any AMD (n = 124)	P value*
Age, median years (IQR)	49 (40–55)	68 (64–75)	76 (71–81)	<0.001
Female, %	64.9	54.1	57.3	0.684
Tobacco use, %				0.404
- Never	45.6	41.0	36.3	
- Former	40.4	42.6	45.2	
- Current	14.0	16.4	18.5	
BMI, median kg/m^2^ (IQR)	24.9 (22.9–28.4)	26.1 (23.0–28.1)	25.7 (23.1–29.1)	0.844
CFH^Y402H^ genotype, %				<0.001
- YY	45.6	39.3	16.9	
- YH	42.1	47.5	43.5	
- HH	12.3	13.1	39.5	

AMD, age-related macular degeneration; IQR, inter quartile range; BMI, body mass index; CFH; complement factor H.

* P value, controls = /> 60y vs Any AMD.

### No change in plasma PTX3 with AMD, sex, age or CFH^Y402H^ genotype

**Fig 1:**
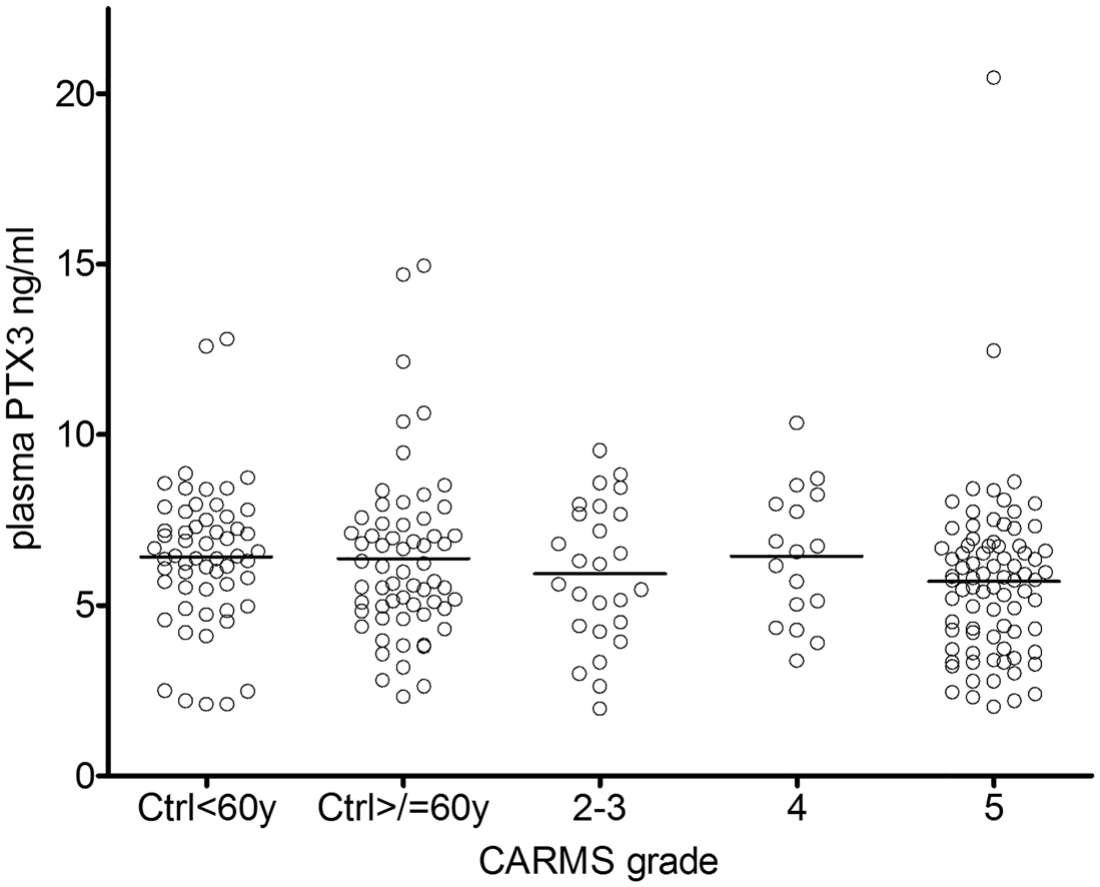
Plasma levels of PTX3 do not differ between AMD patients and controls. PTX3 protein was quantified in plasma samples from patients with AMD (CARMS grade 2–5) and controls (< or >/ = 60 years of age) using ELISA. CARMS grade 2–3, early AMD; 4, geographic AMD; 5, exudative AMD. P = 0.307 in a one-way ANOVA.

Mean (±standard deviation) plasma PTX3 levels were 6.43±2.07 ng/ml for young controls, 6.37±2.49 ng/ml for aged controls, 5.93±2.07 ng/ml for patients with early AMD (CARMS grade 2–3), 6.45±1.96 ng/ml for patients with geographic atrophy (CARMS grade 4), and 5.70±2.50 ng/ml for patients with exudative AMD (CARMS grade 5). All samples analyzed were within the range of the assay and above 1.97 ng/ml. There was no statistically significant correlation with age or disease status (P = 0.307, Fig 1). There was no statistically difference between males and females (data not shown). There was a tendency towards slightly lower plasma PTX3 in patients with exudative AMD compared with controls, but this was not statistically significant. There was no statistical correlation between CFH^Y402H^ genotype and plasma PTX3 (P = 0.055, data not shown), or between plasma levels of CRP and PTX3 (slope: -0.002 +/-0.020, data not shown).

### PTX3 gene expression in human RPE/choroid

**Fig 2:**
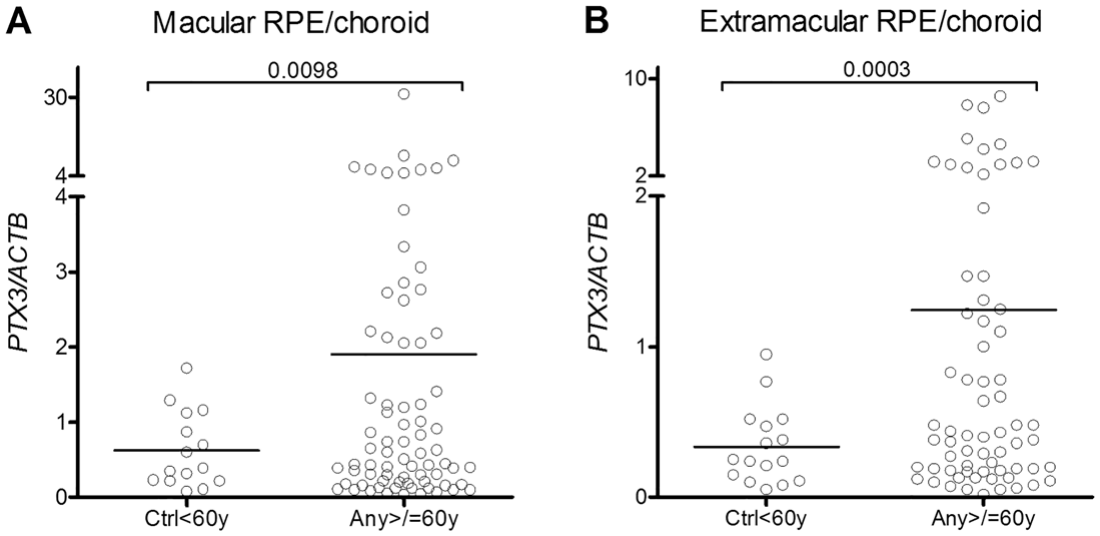
RPE/choroid gene expression of *PTX3* is increased with age. Data were extracted from a publicly available gene expression data set in the NCBI GEO database. *PTX3* gene expression is shown relative to *beta actin (ACTB)* gene expression. Values on bars indicate P values in unpaired t tests with Welch’s correction for unequal variances. Ctrl<60y comprise healthy controls aged 59 or younger (macular, n = 15; extramacular, n = 16). Any >/ = 60y comprise controls (macular, n = 35; extramacular, n = 30) and patients with AMD aged 60 or older (macular, n = 40; extramacular, n = 37).

To investigate whether PTX3 was expressed locally in the retinal tissues, we performed an in silico analysis of publicly accessible gene expression data. Newman et al. [30] recently performed full genome expression analyses of neuroretina and RPE/choroid tissue samples from the macular and extramacular regions of AMD and control donor eyes. We analyzed the combined Iowa/Oregon cohorts for ocular expression of *PTX3*. There was little or no neuroretinal expression of *PTX3* in patients or controls (data not shown). In RPE/choroid samples we found high *PTX3* gene expression. The mean expression levels ranged from 62–236% (macular samples) and 34–149% (extramacular samples) of beta actin gene expression (Fig 2). The *PTX3* expression was increased with age (P = 0.0098 for macular expression and P = 0.0003 for extramacular RPE/choroid), but there was no effect of AMD status (S1 Fig) or sex (data not shown).

### PTX3 expression in ARPE-19 cells

**Fig 3:**
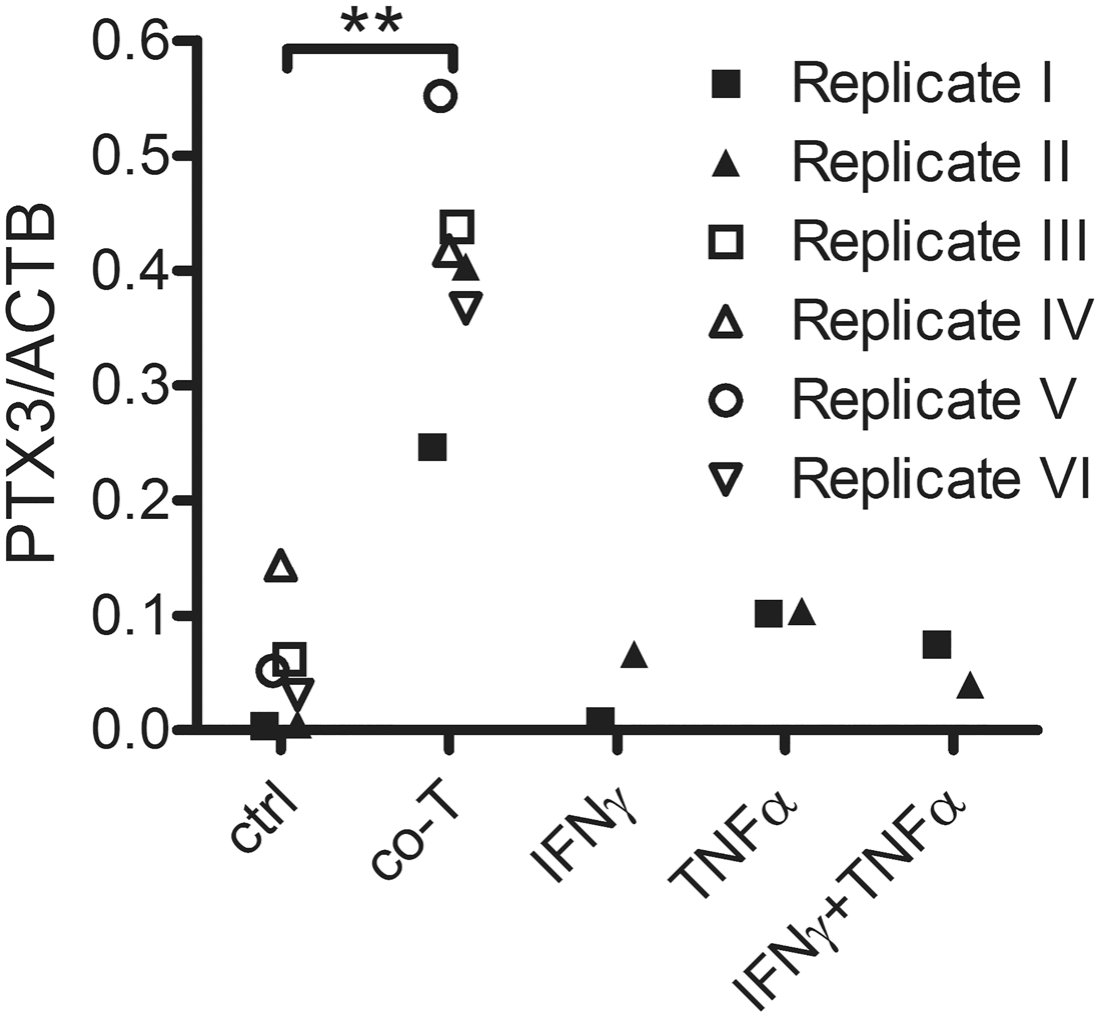
*PTX3* gene expression is increased in ARPE-19 cells following co-culture with activated T cells. Pigmented monolayers of ARPE-19 cells grown on membrane inserts were basolaterally (replicate I-IV) or apically (replicate V-VI) exposed to CD3/CD28-activated human T cells (co-T), recombinant interferon γ (IFNγ) or tumor necrosis factor α (TNFα). RNA was purified and gene expression analyzed using microarrays. **, P<0.01 in one-way ANOVA with Dunnett’s multiple comparison.

**Fig 4:**
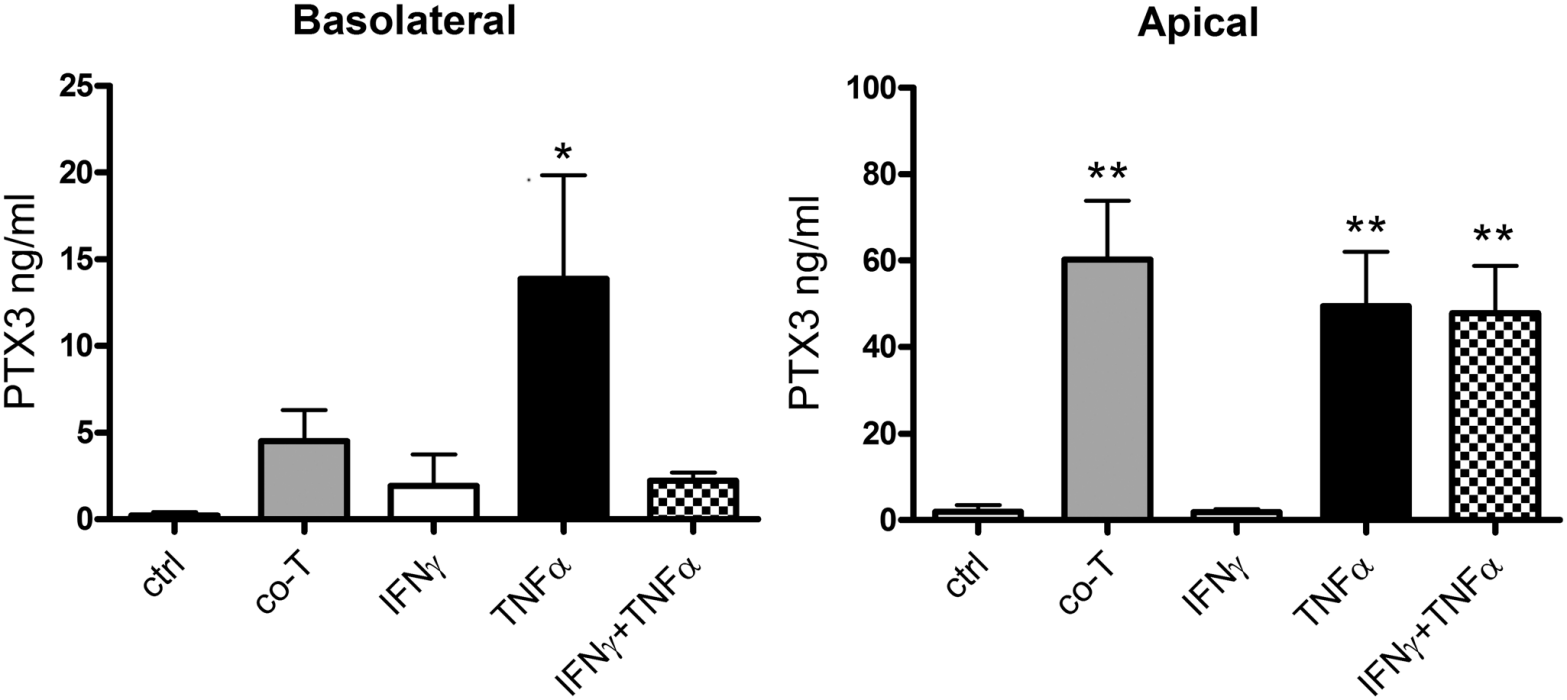
PTX3 secretion from ARPE-19 cells is primarily increased in the apical direction following basolateral inflammatory treatment. Pigmented monolayers of ARPE-19 cells grown on membrane inserts were exposed to CD3/CD28-activated human T cells (co-T) or recombinant cytokines basolaterally. Media was collected from apical and basolateral compartments, and PTX3 was quantified using ELISA. *, P<0.05; **, P<0.01 in repeated measures one-way ANOVA with Dunnett’s multiple comparison. Bars represent mean values from seven independent setups, error bars represent standard error of the mean.

In an effort to further understand factors influencing PTX3 expression from RPE cells, we treated polarized ARPE-19 monolayers with various inflammatory stressors in a transwell system. In a small gene expression screen using microarrays (Fig 3), ARPE-19 were exposed to activated T cells apically or basolaterally. This resulted in significantly increased gene expression of *PTX3* regardless of the direction of stimulation (n = 6; P<0.01). ARPE-19 were also basolaterally exposed to IFNγ and TNFα either alone or in combination (n = 2), but no significant difference in *PTX3* gene expression was noted. To investigate if the observed increase in gene expression translated into increased protein secretion, we treated polarized ARPE-19 monolayers with the same inflammatory stressors from the basolateral compartments (n = 7). We found increased secretion of PTX3 in the apical direction following co-culture with activated T cells or treatment with TNFα with or without IFNγ (P<0.01). The basolateral secretion of PTX3 was also increased after stimulation, but to a lower level and only significantly after treatment with TNFα (Fig 4).

## Discussion

We investigated plasma PTX3 protein levels in AMD patients and controls. We found a higher PTX3 plasma concentration than previously reported was found, but no association with age or AMD status. In RPE/choroid from donor eye tissue, we found no difference in *PTX3* gene expression between aged controls and patients with AMD, but noted an increased expression with age. In vitro, we found increased gene expression and primarily apical protein secretion of PTX3 from RPE cells treated with activated T cells or TNFα. The latter supports a recent study by Woo and co-workers that found PTX3 expression to be increased in ARPE-19 cells following treatment with TNFα or IL1β, but not by IFNγ [19]. These data propose a local role for PTX3 in the aging eye, which is not reflected systemically. We are not aware of studies on PTX3 levels in the vitreous from patients with AMD. However a recent study reported elevated levels of PTX3 in vitreous from patients with central retinal vein occlusion compared to vitreous from patients undergoing surgery for macular hole [33]. This substantiates that ocular PTX3 levels increase under certain conditions and is in line with the observation of apically secreted PTX3 from the RPE. Further studies should examine the in vivo protein expression of PTX3 in RPE/choroid and in the vitreous from control and AMD eyes. AMD pathogenesis is characterized by the formation of drusen, sub-RPE protein/lipid aggregates that may originate from apoptotic cells, cellular blebs or secretions. The major tissue mechanism to clear this debris is based on opsonization by complement factors, resulting in attraction of phagocytes and clearance. The emergence of drusen indicates increased formation of debris or decreased opsonization and clearance, or both.

Dysfunction of the soluble complement regulator, CFH, is suspected to play a key role in AMD pathogenesis. During conditions of inflammation CRP is released from the liver resulting in increased systemic levels. CRP binds to apoptotic cells and CFH, thereby spatially directing the regulatory activity of CFH to meet the increased demand for complement regulation when CRP levels rise. Indeed, increased plasma levels of CRP [34] and complement activating components [35–38] have been correlated to AMD incidence or progression. Conversely, high plasma levels of CFH have been associated with protection from AMD [37,39].

The CFH^402H^ variant, which is found more frequently in AMD patients, exhibits reduced binding to CRP, Bruch’s membrane and other adducts of components of stressed RPE cells [12,40–42]. This reduced CFH binding could result in stronger complement activation against the attacked cell surface. Indeed, carriers of CFH risk variants appear to have systemically increased complement activation and oxidative stress markers [36,38,43], and CFH^402H^ homozygosity combined with increased plasma CRP or high levels of circulating CD56^+^ T cells results in greatly increased risk of AMD [26,44]. These findings indicate that systemic immune activation takes place in AMD, but the causality is unclear. Additionally, the reduced CFH binding to phagocytes has been associated with a higher release of TNFα [45]; when released in the back of the eye, this may result in a higher synthesis of PTX3 in the RPE.

While CRP has been found in the RPE/choroid, it is not produced by the eye. In contrast, PTX3 is a widely expressed protein, which is produced locally in tissues in response to inflammatory stimuli such as cytokines (IL1β, TNFα) and microbial components. Like CRP, PTX3 binds to cell surfaces, and regulates complement activation by attracting CFH and C1q as well as the ficolins. Though the binding of PTX3 has no direct effect on CFH function, the increased anchoring of CFH to cell surfaces effectively increases CFH functions of complement inhibition and iC3b opsonization locally. This could prevent tissue damage caused by excessive complement activation, and assist in clearance of apoptotic cells and debris. Interestingly, PTX3 binds both CFH^Y402H^ variants equally, and may thus play a more important role for CFH regulation in risk-genotype individuals with decreased CRP binding [16].

The relative contributions to ocular complement activation and regulation by systemic and local sources are currently disputed. However, a recent study of liver transplant patients showed that the recipient’s CFH genotype, not the donor’s, was determinant for AMD status five years after transplantation. This indicates that it is indeed the ocular CFH production that is important for AMD pathogenesis. Further, studies showing no association between the CFH^Y402H^ variants and other major diseases with inflammatory components [46–48], indicate that the major impact of the CFH^Y402H^ variant is localized to the eye. These reports support the hypothesis that in AMD, local complement (dys)regulation is mediated by ocularly produced factors.

In conclusion, the results presented in this paper support the notion of an ocular origin of complement factors implicated in AMD. While we did not find any difference in plasma concentrations of PTX3 in AMD patients and controls, we found substantial gene expression of *PTX3* in RPE/choroid tissue, and significantly increased primarily apical secretionof PTX3 in RPE cells following inflammatory stimulation in vitro. This suggests that the RPE reacts to inflammatory stress by increasing expression of PTX3, likely resulting in subretinal increases in PTX3 concentration. It remains to be determined, if induction of PTX3 in RPE could lead to a beneficial, increased CFH-mediated complement regulation and debris opsonization, and in turn, to increased debris removal and a decreased rate of drusen synthesis.

## Supporting Information

S1 Fig The *PTX3* gene expression in RPE/choroid is not correlated to AMD status. Data were extracted from a publicly available gene expression data set in the NCBI GEO database. *PTX3* expression is shown relative to *beta actin (ACTB)* gene expression. Values on bars indicate P values in unpaired t tests with Welch’s correction for unequal variances. Ctrl<60y comprise healthy controls aged 59 or younger (macular, n = 15; extramacular, n = 16). Ctrl >/ = 60y comprise controls aged 60 or older (macular, n = 35; extramacular, n = 30). AMD >/ = 60y comprise patients with AMD aged 60 or older (macular, n = 40; extramacular, n = 37).
(TIF)
