# Supplementary figures and images for: Systemic and Ocular Long Pentraxin 3 in Patients with Age-Related Macular Degeneration
Source: PLoS One. 2015 Jul 15;10(7):e0132800. doi: 10.1371/journal.pone.0132800 (PMC4503310; doi:10.1371/journal.pone.0132800)

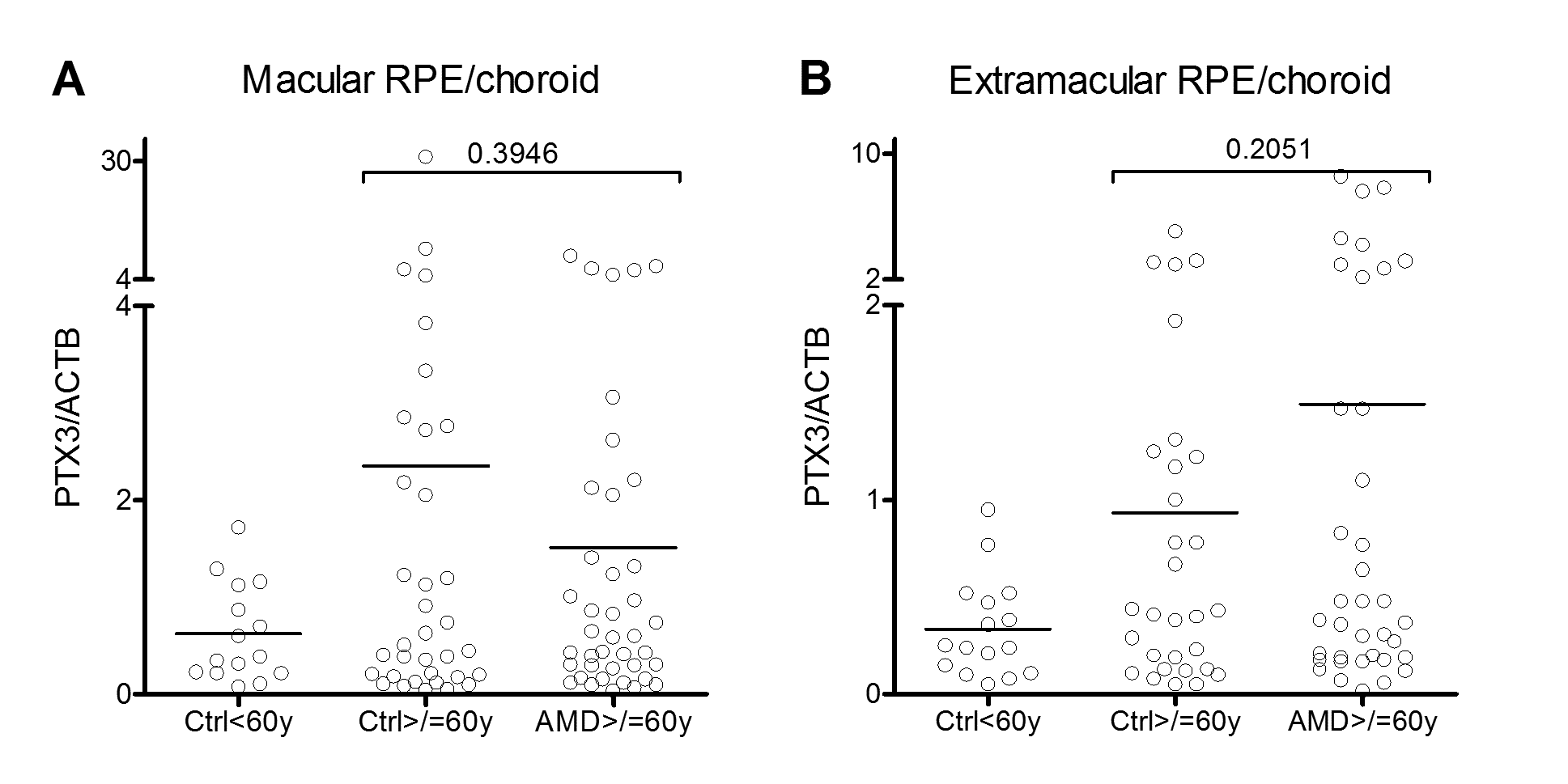

Supplement: S1 Fig — Data were extracted from a publicly available gene expression data set in the NCBI GEO database. PTX3 expression is shown relative to beta actin (ACTB) gene expression. Values on bars indicate P values in unpaired t tests with Welch’s correction for unequal variances. Ctrl<60y comprise healthy controls aged 59 or younger (macular, n = 15; extramacular, n = 16). Ctrl >/ = 60y comprise controls aged 60 or older (macular, n = 35; extramacular, n = 30). AMD >/ = 60y comprise patients with AMD aged 60 or older (macular, n = 40; extramacular, n = 37). (TIF) [file pone.0132800.s001.tif]
